# A meta‐analysis of the effects of synchronization protocols applied to sheep in Turkey on pregnancy rates during breeding and non‐breeding seasons

**DOI:** 10.1002/vms3.610

**Published:** 2021-08-17

**Authors:** Mehmet Saltuk Arikan, Burak Mat, Hasan Alkan, Mustafa Bahadır Çevrimli, Ahmet Cumhur Akin, Tuğba Sarıhan Şahin, Mustafa Agah Tekindal

**Affiliations:** ^1^ Department of Animal Health Economics and Management, Faculty of Veterinary Medicine Fırat University Elazıg Turkey; ^2^ Department of Animal Health Economics and Management, Faculty of Veterinary Medicine Selçuk University Konya Turkey; ^3^ Department of Obstetrics and Gynaecology, Faculty of Veterinary Medicine Selcuk University Konya Turkey; ^4^ Department of Animal Health Economics and Management, Faculty of Veterinary Medicine Mehmet Akif Ersoy University Burdur Turkey; ^5^ Department of Animal Health Economics and Management, Faculty of Veterinary Medicine Ankara University Ankara Turkey; ^6^ Department of Biostatistics, Faculty of Medicine İzmir Katip Çelebi University İzmir Turkey

**Keywords:** breeding season, estrus synchronization, ewes, meta‐analysis, reproductive management

## Abstract

This study aimed to determine common pregnancy rates and effect sizes with meta‐analysis of studies investigating the effect of different synchronization protocols applied to sheep during breeding and non‐breeding seasons on pregnancy rates. Common pregnancy rates were estimated by coalescing pregnancy rates of studies performed independently, and heterogeneity between the studies was investigated. The meta‐analysis included 24 studies that determined pregnancy rates in 78 different groups consisting of 1934 sheep with five different synchronization protocols in Turkey between 2001 and 2020. Among the different synchronization methods, the P4+PMSG group (90.37%) during the breeding season and P4+PGF2α (69.77%) and P4 (68.75%) groups during the non‐breeding season showed the highest pregnancy rate. Also, the effect size of P4+PMSG application during the breeding season was calculated as 0.934 (95% confidence interval: 0.901–0.967), and the effect size of P4+PGF2α application during the non‐breeding season was calculated as 0.709 (95% confidence interval: 0.406–1.013). To conclude, the combination of P4+PMSG during the breeding season and progestogen and other hormone applications during the non‐breeding season are the most effective methods for estrus synchronization and for achieving the desired pregnancy rates.

## Graphical abstract

Pregnancy rates of sheep during breeding and non‐breeding seasons. The effect of different synchronization methods applied to sheep. Reproductive management of sheep breeding in Turkey

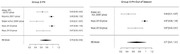

## 1 INTRODUCTION

Estrus synchronization in sheep and goats allows for conducting breeding in livestock farms according to a determined plan, completing it collectively and in a short time, performing births at the desired time, using feed resources, shelter, and workforce more efficiently, and determining the prices of animal products of the enterprise according to market standards (Whitley & Jackson, 2004).

The mating process in sheep and goats is a physiological phenomenon that is highly dependent on the season, and sheep and goats regularly show estrus (polyestrous) during the mating season until pregnancy occurs. Aside from the mating season, sheep and goats enter a resting phase during which sexual activity does not occur, and this period is called anestrus (Öztürkler, 2015). Because breeding activities in sheep and goats depend on seasons, estrus synchronization methods differ by season. The breeding season in the northern hemisphere, in which Turkey is also located, begins in the late summer as the days start to shorten and continues until the end of autumn and early winter (İbiş & Ağaoğlu, 2016).

Various hormone applications are used in estrus synchronization to control breeding in sheep. To this end, hormones such as progestogens, PGF_2α_ and its analogues, pregnant mare's serum gonadotropin (PMSG or eCG) and gonadotropin‐releasing hormone (GnRH), and melatonin, are administered alone or in combination. Progestogens are used during and outside the breeding season, PGF_2α_ and its analogues are used during the breeding season, and melatonin is typically used outside the breeding season (Kaçar et al., 2016).

Many studies have been carried out to investigate the effect of various synchronization applications in sheep on pregnancy rates during breeding and non‐breeding seasons. A wide distribution of pregnancy rates obtained from these studies clearly reveals the necessity of reaching more precise results, and one of the effective methods used to achieve this end is meta‐analysis.

This study aimed to determine common pregnancy rates and effect sizes with a meta‐analysis of studies investigating the effect of different synchronization methods applied to sheep in Turkey during breeding and non‐breeding seasons on pregnancy rates.

## 2 MATERIALS AND METHODS

In this study, subgroups were formed according to different synchronization methods affecting the pregnancy rate in sheep during breeding and non‐breeding seasons. The control groups used in the studies constituted the first group, and melatonin, progestogen (P4), P4+PGF_2α_, P4+PMSG, and PGF_2α_ groups constitute the second, third, fourth, fifth, and sixth groups, respectively. Each group, which was created for meta‐analysis, was examined according to the pregnancy status both in the breeding season and outside the breeding season.

The material of this study consists of pregnancy rates of 78 groups obtained from 24 studies conducted in Turkey between 2001 and 2020 by using five different synchronization methods in sheep during breeding and non‐breeding seasons.

### 2.1 Literature review

**FIGURE 1:**
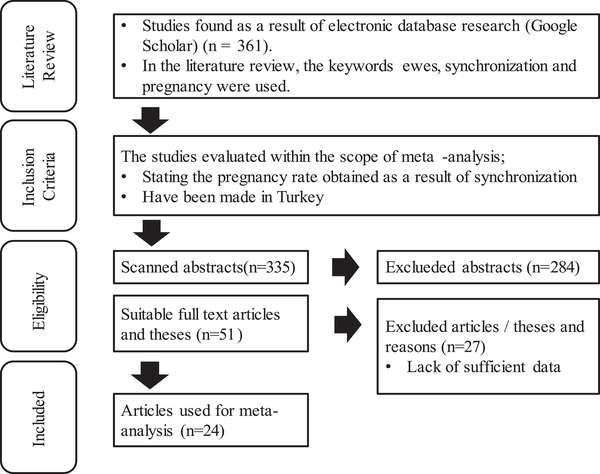
Flow chart on the inclusion criteria of studies in meta‐analysis

Within the scope of the study, a total of 361 studies were identified following the literature review strategy. Among these studies, the abstracts of 335 articles that remained after excluding duplicated articles per research strategies were read. Based on exclusion and inclusion criteria, 284 articles were excluded and 51 studies remained. Again, according to the research literature search strategy, 27 studies that did not provide the necessary statistical data were excluded, and the remaining 24 studies were analyzed in terms of content and transferred to the coding form. The results of the literature review are shown in the flow chart in Figure 1 (Moher et al., 2009). The meta‐analysis included 1934 sheep from 78 different application groups.

### 2.2 Meta‐analysis

Egger's linear regression test was used to determine whether the effect sizes and standard errors of the studies included in the meta‐analysis were linear. To eliminate publication bias, the trim‐and‐fill method of Duval and Tweedie (2000) was used to recalculate the common exposure value. The random‐effects model (Sidik–Jonkman–Knapp–Hartung method) was used to determine the variance between studies as well as the in‐study variance (IntHout et al., 2014; Knapp & Hartung, 2003; Sidik & Jonkman, 2002). Cochran's Q statistics with (*k* – 1) degrees of freedom was applied to evaluate the heterogeneity of the effect sizes of the studies. *I* ^2^ statistics and *τ* ^2^ statistics were employed to determine the level of heterogeneity and the true variance between studies, respectively. The *I* ^2^ value was evaluated by using three categories (low heterogeneity if below 25%, medium if between 25% and 50%, and high if above 50%) proposed by Patsopoulous et al. (2008). In this study, the *I* ^2^ value was found to be less than 50%.

In agreement with the stratification method, which is the most commonly used method to investigate heterogeneity values, the study evaluated the effect sizes of the selected studies in subgroups according to the synchronization method applied and the season status.

## 3 RESULTS

Meta‐analysis was conducted to consolidate the studies that calculated the pregnancy rates using the synchronization methods in sheep in Turkey. Common pregnancy rates were determined according to the consolidated groups being in and out of season. The properties of the subgroups used in the meta‐analysis are listed in Table 1.

**TABLE 1:** Properties of subgroups formed from studies selected for meta‐analysis

Groups	Synchronization protocol	Season status	Total number of sheep	Number of pregnant sheep	Common pregnancy rate (%)
Group 1	Control	In season	83	63	75–90
		Out of season	176	66	37.50
Group 2	Melatonin	In season	20	17	85.00
		Out of season	50	32	64.00
Group 3	P4	In season	64	53	82.81
		Out of season	48	33	68.75
Group 4	P4+PGF_2α_	In season	278	194	69.78
		Out of season	215	150	69.77
Group 5	P4+PMSG	In season	301	272	90.37
		Out of season	625	371	59.36
Group 6	PGF_2α_	In season	29	23	79.31
		Out of season	45	25	55.56

As shown in Table 1, the highest pregnancy rate during the breeding season (90.37%) and outside the breeding season (69.77%) was obtained from synchronization applications performed using P4+PMSG and P4+PGF_2α_, respectively.

There is a moderate bias in our study that examines the effects of synchronization methods on pregnancy rates in sheep, and Table 2 shows the heterogeneity test statistics of publication bias in the groups formed.

**TABLE 2:** Publication bias summary statistics of studies on the effect of synchronization protocols on pregnancy rates in sheep

	Fail‐safe N analysis (file drawer analysis)		Rank correlation test for funnel plot asymmetry		Regression test for funnel plot asymmetry		Heterogeneity statistics						
	Fail‐safe N	*p*	Kendall's tau	*p*	*Z*	*p*	*τ*	*τ* ^2^	*I* ^2^	*H* ^2^	df	*Q*	*p*
Group 1‐control	2408.000	<0.001	0.257	0.202	1.264	0.206	0.346	0.1196 (SE = 0.0493)	45.76%	23.587	14.000	441.773	<0.001
Group 1‐control‐in‐season	620.000	<0.001	–0.467	0.272	–1.528	0.127	0.042	0.0018 (SE = 0.0085)	12.34%	1.144	5.000	5.746	0.032
Group 1‐control‐out of season	577.000	<0.001	0.389	0.180	0.477	0.633	0.339	0.115 (SE = 0.0607)	46.73%	30.559	8.000	326.308	<0.001
Group 2‐melatonin	377.000	<0.001	–0.333	0.750	–1.465	0.143	0.312	0.0974 (SE = 0.0861)	42.55%	13.417	3.000	32.568	<0.001
Group 2‐melatonin‐in season													
Group 2‐melatonin‐out of season	167.000	<0.001	–0.333	0.999	–0.939	0.348	0.365	0.1335 (SE =)	44.01%	16.684	2.000	29.219	<0.001
Group 3‐P4	1560.000	<0.001	–0.600	0.233	–5.003	<0.001	0.206	0.0424 (SE = 0.0353)	41.24%	11.412	4.000	30.538	<0.001
Group 3‐P4‐in season													
Group 3‐P4‐out of season	365.000	<0.001	–0.333	0.999	–2.229	0.026	0.249	0.0621 (SE = 0.0718)	48.02%	8.349	2.000	21.064	<0.001
Group 4‐P4+PGF	5017.000	<0.001	–0.242	0.311	–1.219	0.223	0.092	0.0085 (SE = 0.0063)	43.07%	2.708	11.000	25.181	0.009
Group 4‐P4+PGF‐in season	891.000	<0.001	–0.333	0.750	0.146	0.884	0.070	0.0049 (SE = 0.0073)	49.02%	2.440	3.000	5.256	0.004
Group 4‐P4+PGF‐out of season	1673.000	<0.001	–0.571	0.061	–2.141	0.032	0.111	0.0122 (SE = 0.0107)	43.86%	2.767	7.000	19.568	0.007
Group 5‐P4+PMSG	89,812.000	<0.001	–0.404	<.001	–1.501	0.133	0.210	0.0442 (SE = 0.0119)	43.36%	15.067	37.000	546.022	<0.001
Group 5‐P4+PMSG‐in season	27,844.000	<0.001	–0.642	<.001	–4.423	<0.001	0.034	0.0012 (SE = 0.0017)	29.99%	1.428	17.000	22.733	0.015
Group 5‐P4+PMSG‐out of season	17,625.000	<0.001	–0.305	0.064	–0.994	0.320	0.260	0.0675 (SE = 0.0238)	44.46%	18.055	19.000	376.376	<0.001
Group 6‐PGF	408.000	<0.001	–0.333	0.750	0.358	0.720	0.366	0.1339 (SE = 0.1154)	45.67%	23.075	3.000	95.589	<0.001
Group 6‐PGF‐in season													
Group 6‐PGF‐out of season													

As shown in Table 2, the meta‐analysis of the studies included in this study was found to be heterogeneous because the *p*‐value was <0.05, and the *Q* value was greater than the value corresponding to the df value as a result of the heterogeneity test.

As the statistical values of *I* ^2^ we used to determine the level of heterogeneity were found to be below 50%, it can be concluded that the study involves a moderate bias and, therefore, the random‐effects model was chosen. Table 3 shows the distribution value, average effect size, and confidence intervals of the random‐effects model.

**TABLE 3:** Statistical values of the random‐effects model of synchronization protocols in sheep

	Random‐effects model						
		Estimate	SE	*Z*	*p*	CI lower bound	CI upper bound
Group 1‐control	Intercept	0.459	0.0932	4.92	<0.001	0.276	0.642
Group 1‐control‐in‐season	Intercept	0.789	0.0474	16.6	<0.001	0.696	0.882
Group 1‐control‐out of season	Intercept	0.285	0.116	2.46	0.014	0.058	0.513
Group 2‐melatonin	Intercept	0.691	0.162	4.26	<0.001	0.373	1.009
Group 2‐melatonin‐in season	Intercept						
Group 2‐melatonin‐out of season	Intercept	0.636	0.218	2.92	0.003	0.209	1.063
Group 3‐P4	Intercept	0.755	0.1000	7.55	<0.001	0.559	0.950
Group 3‐P4‐in season	Intercept						
Group 3‐P4‐out of season	Intercept	0.709	0.155	4.59	<0.001	0.406	1.013
Group 4‐P4+PGF	Intercept	0.710	0.0351	20.2	<0.001	0.641	0.779
Group 4‐P4+PGF‐in season	Intercept	0.712	0.0474	15.0	<0.001	0.620	0.805
Group 4‐P4+PGF‐out of season	Intercept	0.705	0.0499	14.1	<0.001	0.607	0.802
Group 5‐P4+PMSG	Intercept	0.780	0.0367	21.3	<0.001	0.708	0.851
Group 5‐P4+PMSG‐in season	Intercept	0.934	0.0167	56.0	<0.001	0.901	0.967
Group 5‐P4+PMSG‐out of season	Intercept	0.694	0.0605	11.5	<0.001	0.575	0.812
Group 6‐PGF	Intercept	0.649	0.188	3.45	<0.001	0.280	1.017
Group 6‐PGF‐in season	Intercept						
Group 6‐PGF‐out of season	Intercept						

*Note*: *τ* ^2^ estimator: Empirical Bayes.

As shown in Table 3, the effect size (0.780) of the synchronization protocol applied using P4+PMSG in Group 5 is larger than the effect size of the groups formed by the other protocols. When a comparison was made in terms of the season, it was found that the effect size (0.934) of the in‐season applications in Group 5 was larger than the out‐of‐season effect size (0.694).

It has also been observed that synchronization works performed in sheep in Turkey during the determined period (2001–2020) had a significant effect on pregnancy rates, and hormone applications performed to increase pregnancy rates significantly increased this value statistically.

The effect sizes of in‐season and out‐of‐season applications in subgroups created for meta‐analysis in the study are discussed in what follows.

**FIGURE 2:**
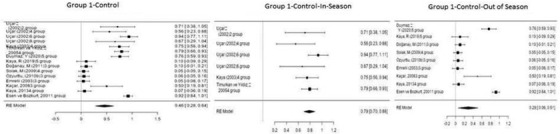
Forest plot showing the impact direction of studies in Group 1

Figure 2 shows the forest plot obtained as a result of the meta‐analysis applied to Group 1 (control).

Figure 2 provides a summary of the effect sizes and relative weights of each study with the findings of the forest plot. The squares on the left in the forest plot show the effect size of each study, the sizes of the squares show the study sizes, and the bars extending to the right and left show, respectively, the lower and upper limit of the 95% confidence interval of each study's effect size. The diamond at the *x*‐axis in the plot shows the overall effect size, and the overall effect size is found to be 0.79 (95% confidence interval: 0.70–0.88) in the in‐season control group and 0.29 (95% confidence interval: 0.06–0.51) in the out‐of‐season group (*p* < 0.001).

**FIGURE 3:**
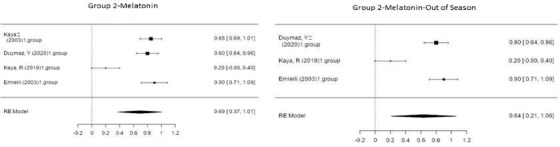
Forest plot showing the impact direction of studies in Group 2

Figure 3 shows the forest plot obtained as a result of the meta‐analysis applied to the synchronization studies conducted using melatonin in Group 2.

As shown in Figure 3, the highest effect size (0.90) was found in the group used in the study by Emrelli et al. (2003), where the off‐season synchronization studies were performed using melatonin. In this group, 18 mg of melatonin was administered as a behind‐the‐ear implant to sheep in off‐season anestrus, and a pregnancy rate of 90% was determined (Emrelli et al., 2003).

**FIGURE 4:**
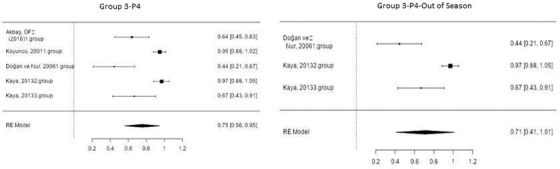
Forest plot showing the impact direction of studies in Group 3

Figure 4 shows the forest plot obtained as a result of the meta‐analysis applied to the synchronization studies conducted using P4 in Group 3.

As indicated in Figure 4, the highest effect size (0.97) was noted in the group used by Kaya (2013) in the off‐season synchronization studies conducted using progesterone. In this group, 1000 IU of hCG was injected into sheep intramuscularly 7 days after sponge application (20 mg of fluorogestone acetate) during the non‐breeding season, and a pregnancy rate of 100% was reported (Kaya, 2013).

**FIGURE 5:**
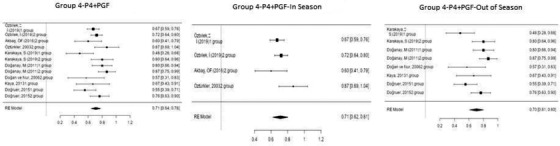
Forest plot showing the impact direction of studies in Group 4

Figure 5 shows the forest plot obtained as a result of the meta‐analysis applied to the synchronization studies conducted using P4+PGF in Group 4.

As reported in Figure 5, the highest effect size (0.87) was observed in the group used by Öztürkler et al. (2003) in the in‐season synchronization studies conducted using P4+PGF (Öztürkler et al., 2003). In this group, 0.075 mg of cloprostenol was injected into sheep intramuscularly 5 days after intravaginal sponge application during the breeding season, and a pregnancy rate of 86.7% was achieved. In off‐season synchronization, Doğanay (2011) placed intravaginal sponges in sheep for 14 days and then administered 400 IU of PMSG intramuscularly to sheep on the day the sponges were removed, and a pregnancy rate of 86.6% was realized (Doğanay, 2011).

**FIGURE 6:**
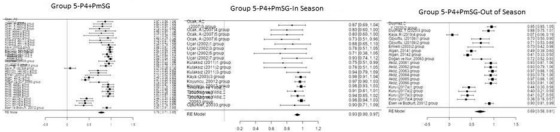
Forest plot showing the impact direction of studies in Group 5

Figure 6 shows the forest plot obtained as a result of the meta‐analysis applied to the synchronization studies conducted using P4+PMSG in Group 5.

As shown in Figure 6, the highest effect size (0.98) was found in the groups used by Timurkan and Yildiz (2005) and Koyuncu et al. (2001) in the in‐season synchronization studies conducted using P4+PMSG. Timurkan and Yildiz (2005) and Koyuncu et al. (2001) placed intravaginal sponges in sheep for 14 days, then, respectively, administered 750 and 700 IU of PMSG intramuscularly to sheep on the day the sponges were removed, and finally achieved a pregnancy rate of 100%.

Outside the season, 500 and 700 IU of PMSG were injected into sheep 7 days after the application of intravaginal sponge in a different group, and a pregnancy rate of 100% was achieved, and the effect size of these studies was found to be 0.97 during the meta‐analysis (Aköz et al., 2006).

**FIGURE 7:**
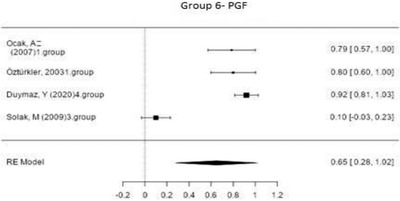
Forest plot showing the impact direction of studies in Group 6

Figure 7 shows the forest plot obtained as a result of the meta‐analysis applied to the synchronization studies conducted using PGF in Group 6.

As indicated in Figure 7, the highest effect size (0.92) was found in the group used by Duymaz (2020) in the in‐season synchronization studies conducted using PGF (Duymaz, 2020). In this group, 3 cc of prostaglandin was injected into sheep intramuscularly in two doses at 11‐day intervals during the breeding season. Then, a pregnancy rate of 92% was reported.

## 4 DISCUSSION AND CONCLUSION

In addition to meta‐analysis being a method that combines and summarizes independent and comparable studies, it summarizes the effect sizes obtained from each study with a single statistic. This analysis allows for eliminating inconsistencies in individual studies to make stronger and more accurate estimates for the effect size of the population. These estimates also find a place in veterinary medicine and are widely applied in this field (Diaz et al., 2019; Palacin et al., 2011; Yan et al., 2016). This study evaluated both the effect of different synchronization methods applied to sheep on pregnancy rates and the in‐season and out‐of‐season status of sheep in each subgroup.

For sheep and goats, melatonin is a vital hormone in initiating a series of reproductive events at the beginning of the breeding season (Abecia et al., 2019). Therefore, estrus synchronization is tried with different applications of melatonin in sheep and goats (Abecia et al., 2007; De Nicolo et al., 2008). This study also evaluated the effect of melatonin applications in sheep during and outside the breeding season in Turkey. In synchronization applications with melatonin, the pregnancy rates achieved during the breeding season were found to be higher than the ones outside the season. However, it was found to be higher than in the off‐season PGF_2α_ application and control groups. In many studies, the pregnancy rates achieved were low in off‐season applications of melatonin. Therefore, it was suggested that it may be more useful to apply melatonin together with hormones such as progesterone and PMSG in off‐season applications (De Nicolo et al., 2008; Kridli et al., 2006). However, the reason for an increase in pregnancy rates during the breeding season is thought to be because melatonin exhibits a luteotropic effect and increases the amount of progesterone and the chance of survival of the embryo (Horoz et al., 2003; Wellace et al., 1988). Also, melatonin supports early corpus luteum and embryo development (Abecia et al., 2019, 2002; Bittman et al., 1985; Horoz et al., 2003).

Progesterone is mostly applied with synchronization protocols in sheep and goats during breeding and non‐breeding seasons (Abecia et al., 2012; Menchaca et al., 2017; Wildeus, 2000). It was also found that progesterone had been mainly used in the majority of synchronization studies performed in Turkey. The use of progestogens alone in these protocols is considered effective in achieving the desired pregnancy rates (Abecia et al., 2012; Menchaca et al., 2017; Skliarov et al., 2021). This study also achieved an average pregnancy rate of 82.81% from the application of P4 alone during the breeding season. On the other hand, although progestogen‐based estrus synchronization protocols are applied alone in many studies conducted in the world and Turkey, the application of progestogens in combination with PMSG or PGF_2α_ is also found to be effective. It is reported that pregnancy rates increase following the P4+PMSG applications, especially during the breeding season. The aim of applying progestogens in estrus synchronization is to suppress the release of gonadotropin and stimulate ovarian activity through PMSG administered at the end of the application. The purpose of this is to imitate the estrus cycle for increasing the rate of pregnancy (Abecia et al., 2012; Koyuncu & Ozis Alticekic, 2010; Menchaca et al., 2017; Ramos and Silva, 2018).

Indeed, when the studies conducted in Turkey were examined, it was observed that the highest pregnancy rates during the breeding season were achieved following the P4+PMSG applications. However, when the progesterone‐based estrus synchronization protocols performed outside the breeding season were studied, the rates of pregnancy were found to be lower than during the breeding season. The factors that result in the low pregnancy rates in off‐season applications are the animals being in deep anestrus, decreased hormonal effects, and low ovarian activity.

As shown in Tables 2 and 3, the meta‐analysis of the studies included in the study was found to be heterogeneous because the *p*‐value was <0.05 and the *Q* value was greater than the value corresponding to the df value as a result of the heterogeneity test. As the statistical values of *I* ^2^ we used to determine the level of heterogeneity were found to be below 50%, the study involves a moderate bias, and therefore, the random‐effects model was chosen. Although the average result is estimated to be low, the actual result in some studies may actually be positive.

With estrus synchronization in sheep breeding, pregnancy is controlled during both breeding and non‐breeding seasons. In sheep breeding, pregnancy planning under operating conditions aims to provide an optimum yield by spreading the lamb or milk yield to the whole year in line with the purpose of the enterprise. Also, it is highly possible to control reproductive performance with synchronization protocols in sheep. However, the methods that provide optimum success by achieving economic efficiency at the same time under operating conditions should be explored. To this end, the meta‐analysis performed by consolidating the results of this study and the results of studies conducted on the effects of synchronization protocols on in‐season and off‐season pregnancy rates in sheep serves as a guide and provides a decision support system to achieve the target success in enterprises. To conclude, it is found, according to the studies conducted in Turkey, that the P4+PMSG application is the most effective method for achieving estrus synchronization and the desired pregnancy rates during the breeding season and the combined application of progestogens and other hormones is found to be effective during the non‐breeding season.

## CONFLICT OF INTEREST

The authors declare no conflict of interest.

### PEER REVIEW

The peer review history for this article is available at https://publons.com/publon/10.1002/vms3.610
